# Supply of Neuraminidase Inhibitors Related to Reduced Influenza A (H1N1) Mortality during the 2009–2010 H1N1 Pandemic: An Ecological Study

**DOI:** 10.1371/journal.pone.0043491

**Published:** 2012-09-11

**Authors:** Paula E. Miller, Aksharananda Rambachan, Roderick J. Hubbard, Jiabai Li, Alison E. Meyer, Peter Stephens, Anthony W. Mounts, Melissa A. Rolfes, Charles R. Penn

**Affiliations:** 1 Department of Statistics, Saint Olaf College, Northfield, Minnesota, United States of America; 2 IMS Health, London, United Kingdom; 3 World Health Organization, Geneva, Switzerland; 4 Division of Epidemiology and Community Health, School of Public Health, University of Minnesota, Minneapolis, Minnesota, United States of America; University of Ottawa, Canada

## Abstract

**Background:**

The influenza A (H1N1) pandemic swept across the globe from April 2009 to August 2010 affecting millions. Many WHO Member States relied on antiviral drugs, specifically neuraminidase inhibitors (NAIs) oseltamivir and zanamivir, to treat influenza patients in critical condition. Such drugs have been found to be effective in reducing severity and duration of influenza illness, and likely reduced morbidity during the pandemic. However, it is less clear whether NAIs used during the pandemic reduced H1N1 mortality.

**Methods:**

Country-level data on supply of oseltamivir and zanamivir were used to predict H1N1 mortality (per 100,000 people) from July 2009 to August 2010 in forty-two WHO Member States. Poisson regression was used to model the association between NAI supply and H1N1 mortality, with adjustment for economic, demographic, and health-related confounders.

**Results:**

After adjustment for potential confounders, each 10% increase in kilograms of oseltamivir, per 100,000 people, was associated with a 1.6% reduction in H1N1 mortality over the pandemic period (relative rate (RR) = 0.84 per log increase in oseltamivir supply). While the supply of zanamivir was considerably less than that of oseltamivir in each Member State, each 10% increase in kilogram of active zanamivir, per 100,000, was associated with a 0.3% reduction in H1N1 mortality (RR = 0.97 per log increase).

**Conclusion:**

While there are limitations to the ecologic nature of these data, this analysis offers evidence of a protective relationship between antiviral drug supply and influenza mortality and supports a role for influenza antiviral use in future pandemics.

## Introduction

The 2009 influenza A (H1N1) pandemic provoked large-scale public health responses and implementation of pandemic preparedness plans throughout the world. Clinical trials have shown that neuraminidase inhibitors (NAIs), a class of antiviral drugs including oseltamivir and zanamivir, are efficacious in lowering morbidity related to influenza, reducing both the duration of symptoms from influenza and the overall severity of the illness [1], [2], [3], [4]. Furthermore, modeling studies suggest that treatment of symptomatic individuals with antivirals during a pandemic can reduce the overall disease attack rate and lessen the overall scope of local epidemics [5], [6], [7]. These results prompted public health organizations, such as the World Health Organization (WHO) and the Centers for Disease Control and Prevention (CDC), to recommend antiviral drug treatment of influenza in the event of a pandemic [8], [9]. As such, many WHO Member States ordered and distributed significant amounts of NAIs in order to treat and control the spread of influenza.

Whether that use of NAIs had a meaningful impact on influenza mortality during the pandemic is currently being explored. In general, a recent meta-analysis of observational studies of influenza treatment outside of the 2009 H1N1 pandemic indicated that, on an individual level, there is low-quality, but supportive evidence, that treatment with antivirals, and particularly within 48 hours of symptom onset, is associated with improved survival [10]. During the 2009 H1N1 pandemic, patients in the United Kingdom (UK) treated with antivirals before being admitted to the hospital were 50% less likely to die in the hospital and were also less likely to require admission to the intensive care unit [11]. Additionally, hospitalized patients with confirmed influenza in New York City who survived were more likely to have received oseltamivir within 48 hours of hospitalization than those who died [12]. A retrospective analysis of patients seen during the H1N1 pandemic in Beijing found that 80% of the inpatients evaluated received antiviral treatment and found oseltamivir to be beneficial [13]. However, not all studies have found evidence of a clear benefit. One short observational report from Japan indicated that, despite 80% of fatal cases receiving antivirals, there was no difference in the timing of antiviral treatment between fatal cases and non-fatal but severe cases [14]. In a different cohort from Beijing, no difference in antiviral usage was found between survivors and non-survivors among hospitalized cases; although, antiviral treatment seemed to be delayed in most patients with only 10% of patients receiving treatment within 48 hours of symptom onset [15].

On an ecologic level, wide disparities in rates of NAI supply existed across WHO Member States during the H1N1 pandemic. For example, in France, Germany, and Japan NAIs were widely prescribed for patients exhibiting influenza symptoms [16], [17]. Other Member States, such as Argentina, Spain, and the UK, were much more reserved in prescribing antiviral drugs for treatment of suspected pandemic H1N1 cases [16]. Likewise, a wide range of H1N1-specific mortality across Member States was observed. For example, the mortality rate in Argentina was 1.73 per 100,000 people while in Japan the mortality rate was 0.15 per 100,000 [17], [18]. Although a group-level analysis cannot indicate the efficacy or effectiveness of NAIs on individual-level risk of fatal influenza, it can inform policy makers and community leaders of the impact of an aggregate policy, such as supply of or investment in antivirals, on overall mortality trends during a pandemic.

The purpose of this ecological analysis was, therefore, to examine the relationship of mortality specific to pandemic H1N1 and NAI supply at the level of WHO Member States and provide further evidence of the aggregate role that NAIs may play in reducing influenza mortality in future pandemics.

## Methods

Data for total kilograms of neuraminidase inhibitors (NAIs) distributed and lab-confirmed deaths attributed to pandemic H1N1 from July 2009 to August 2010 were available for 62 WHO Member States in all WHO regions. The time period for analysis was chosen in order to correspond with the time during which H1N1 was classified as a pandemic by the WHO. Data on weekly mortality of lab-confirmed pandemic H1N1 influenza were obtained from the WHO. Weekly mortality totals were summed over the entire pandemic period.

Data on country-level NAI supply were collected by Intercontinental Medical Statistics (IMS) Health, an organization that audits transactions occurring between pharmaceutical manufacturers and purchasing hospitals and retail establishments throughout the world. Additional data from IMS provided an estimate of the market coverage in a Member State and specific sales sectors, such as hospital or private sales that may be excluded from the audit. The NAI supply used in this analysis represents kilograms of active drug and is derived from weighted estimates of market coverage provided by IMS audits. Total population estimates for each Member State, derived from the United Nations Statistics Division, were included to estimate the mortality and NAI supply per 100,000 people.

Additional data on possible confounding factors, at the level of the Member State, were compiled from data published by United Nations agencies, as well as other international organizations. The confounding variables fell into three broad categories: health indicators, health-related indicators, and socio-economic indicators (Table 1). Confounders such as under-five mortality and life expectancy were included as indicators of overall development and health of a Member State. Epidemiologic studies suggest that obesity, pregnancy, and age were risk factors for severe pandemic H1N1 influenza and, thus, data on these factors were gathered and considered [19]. Variables for percent of a population using improved sanitation and water quality standards were included as a measure of sanitation practices and ease of disease spread in a Member State. Adult literacy rate was included as a proxy for overall education and, more specifically, as a proxy for education related to health and infection control. Per capita gross domestic product (GDP) served as an indicator of overall economic position. Finally, per capita health spending, physician density, and hospital bed density were included to measure access to and quantity of medical care within a Member State. The pregnancy rate, the proportion of the population pregnant at any given time, was calculated by multiplying childbirths per 1,000 people by 0.77 (40/52), to account for the average duration of gestation [19].

**Table 1:** Health, health-related, and socioeconomic indicators (references noted).

Health Indicators	Under 5 mortality (per 1,000) [24]
	% of Adult Population with HIV [25], [26]
	Life Expectancy (male, female, overall) [27]
	Obesity Rate as body mass index >30 (male, female) [26], [28]
Health Related Indicators	% Population with Improved Sanitation Quality [26]
	% Population with Water Quality Standards [26]
	Environmental Workers (per 10,000) [29]
	Community Health Workers (per 10,000) [29]
	Government Health Spending per Capita [29]
	Private Health Spending per Capita [29]
	Total Per Capita Health Spending [29]
	% of Infant Population with Hepatitis B Vaccination [24]
	Physician Density (per 10,000) [29]
	Hospital Beds (per 10,000) [29]
	Pregnancy Rate (per 1,000) [30]
Socioeconomic Indicators	% Population >65 years [30]
	Adult Literacy Rate [26]
	% Population in Urban Areas [26]
	Measure of Political Stability [31]
	Per Capita Gross Domestic Product [26]
	Population Density [26]

**Figure 1:**
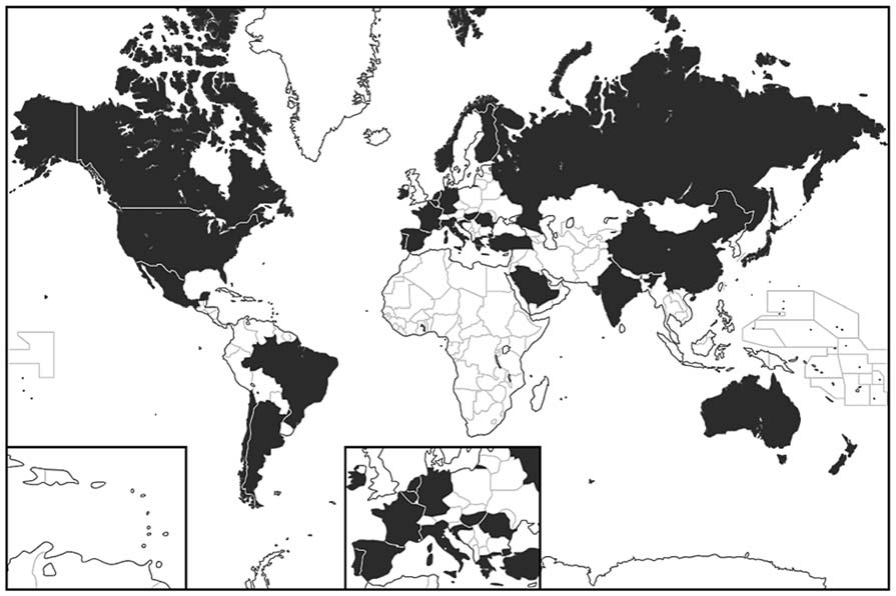
World Health Organization Member States included in analysis (shaded).

Member states were excluded from the model if there was insufficient data available (multiple instances of missing data) related to the identified potential confounding factors. After excluding 20 Member States for data insufficiency, 42 Member States were left for inclusion in the primary analysis (Figure 1).

Poisson regression was used to model H1N1 mortality rate during the 14-month period. Drug supply per capita was log transformed (natural log) in order to meet the normality assumption for regression modeling. Similarly, all confounders were evaluated for normality and were either transformed using the log or 1-log, as needed; briefly, log transformations were applied to the derived pregnancy rate and the density of hospital beds and 1-log transformations were applied to per capita health spending and the adult literacy rate.

To assess confounding, each of the twenty-four possible confounders was individually regressed, after appropriate transformation, against H1N1 mortality using a Poisson model and log-transformed NAI supply per capita using linear regression. Of these, twelve were significantly associated with both pandemic H1N1 mortality rate and NAI rates, and therefore kept as potential confounders for evaluation in multivariable adjusted models. These twelve potential confounders included under five mortality, life expectancy at birth, percent of obese males over the age of 15, percent of population using improved sanitation, percent of population with access to improved water quality standards, derived pregnancy rate, adult literacy rate, percentage of population over the age of 65, per capita gross domestic product (GDP), per capita health spending, physician density, and hospital bed density.

Three models were considered in order to evaluate the effect of NAI supply on pandemic H1N1-specific mortality: the supply of oseltamivir only, the supply of zanamavir only, and a combined model that included both oseltamivir and zanamivir supply. It was found that the combined model most effectively described H1N1 mortality trends; thus, all further results and discussion are based on the combined model.

Backwards stepwise regression methods were used to further reduce the combined models; first starting with a full, saturated model including all twelve potential confounders, and, one at a time, removing covariates that were not significantly associated (had a p-value>0.05) with H1N1 mortality. However, non-significant indicators were retained in the model if it was found that they were still a strong confounder of the NAI and influenza mortality association; namely, that their removal changed the coefficient estimate for NAI supply by more than 10 percent. Model fit criteria, namely the Aikaike information criteria, were also considered when reaching a final model. The final model considered pregnancy rate, life expectancy, hospital bed density, per capita health spending, percentage of the population over 65 years old, under-five mortality rate, adult literacy rate, the rate of male obesity, and physician density. The statistical program R (http://www.r-project.org/) was used for all analysis and p-values≤0.05 were considered statistically significant.

## Results

The median total H1N1-specific mortality rate was 0.65 per 100,000 people among the 42 Member States during the pandemic period from July 2009 to August 2010 (IQR: 0.40–1.05 per 100,000 people). Overall, H1N1-specific mortality peaked in the southern hemisphere during August 2009 and in the northern hemisphere in January 2010. The region of the Americas experienced the highest total H1N1 mortality rates among the regions included in the analysis, while China and Member States in the Eastern Mediterranean regions had much lower mortality during the pandemic (Table 2).

**Table 2:** Median total and monthly estimates of influenza A(H1N1) mortality and kilograms of oseltamivir and zanamivir by World Health Organization region.

		The Americas (AMRO)	China (SEARO)	Europe (EURO)	Eastern Mediterranean (EMRO)	Western Pacific (WPRO)
	Member States included	6	1	26	5	4
**Mortality** *(H1N1 deaths per 100,000 people)*	Total Median	1.18	0.18	0.63	0.37	0.51
	*(IQR)*	(1.08–1.33)	(–)	(0.45–0.87)	(0.15–0.56)	(0.13–0.89)
	Monthly Median	0.084	0.013 (–)	0.045	0.027	0.037
	*(IQR)*	(0.077–0.095)	(–)	(0.032–0.062)	(0.010–0.040)	(0.0096–0.064)
**Oseltamivir** *(kg per 100,000 people)*	Total Median	0.14	0.00013	0.47	0.017	0.46
	*(IQR)*	(0.032–0.46)	(–)	(0.12–0.72)	(0.013–0.57)	(0.29–1.52)
	Monthly Median	0.0097	0.0000094	0.034	0.0012	0.033
	*(IQR)*	(0.0023–0.033)	(–)	(0.0087–0.052)	(0.00090–0.040)	(0.020–0.11)
**Zanamivir** *(kg per 1 million people)*	Total Median	0.0034	0.0002	0.0091	0.024	0.026
	*(IQR)*	(0.00016–0.031)	(–)	(0.0010–0.064)	(0.017–0.037)	(0.015–1.2)
	Monthly Median	0.00024	0.000014	0.00065	0.0017	0.0018
	*(IQR)*	(0.000012–0.0022)	(–)	(0.000072–0.0046)	(0.0012–0.0026)	(0.0011–0.085)

The median total oseltamivir supply over the pandemic period was 0.24 kg of active drug per 100,000 people (IQR: 0.042–0.66), or roughly 1600 adult doses per 100,000, though the range was quite large. The estimated maximum oseltamivir supply over the pandemic was 4.69 kg per 100,000 (31267 doses/100,000) and the minimum was 0.0001 kg (0.7 doses/100,000) per 100,000. For zanamivir, the median supply over the pandemic was much lower at 0.01 kg per million people (IQR: 0.0009–0.05), or roughly 200 adult doses per million. The highest NAI supply rates were seen in the European and Western Pacific Member states, whereas Member States in the Americas and in the Eastern Mediterranean had the lowest total supplies.

**Figure 2:**
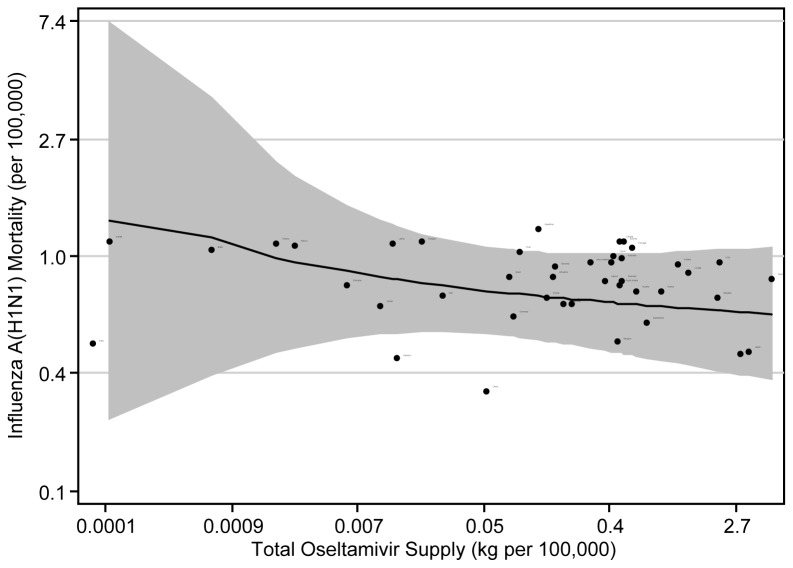
Fitted univariate poisson regression line and observed values for total influenza A (H1N1) mortality, per 100,000 people, by total oseltamivir supply, per 100,000, from April 2009 to August 2010, with corresponding 95% confidence intervals.

In preliminary analysis, without consideration of confounding factors, H1N1-specific mortality during the 14-month pandemic period was negatively related to oseltamivir supply (p-value<0.001, Figure 2). There was also a negative association found between supply of zanamivir and H1N1 mortality, though not as strong as with oseltamivir (p-value<0.001, results not shown). The negative association held in the unadjusted model accounting for both the supply of oseltamivir and zanamivir (results not shown). After adjusting for potential confounders, the final combined model showed a significant negative association between the per capita supply of NAIs and H1N1 mortality. The rate ratio for each log increase in oseltamivir was 0.84 (95% confidence interval (CI): 0.83–0.85) and 0.97 for each log increase in zanamivir (95% CI: 0.96–0.98). In other words, a 10% increase in kilograms of oseltamivir per 100,000 people was associated with a 1.6% decrease in H1N1 mortality rate and a 10% increase in kilograms of zanamivir per 100,000 was associated with a 0.3% decrease in mortality rate.

## Discussion

While antiviral drugs were relied on during the recent H1N1 pandemic, questions remain regarding their effectiveness in reducing influenza, and specifically pandemic H1N1, mortality. Data from the WHO on H1N1 mortality from the recent pandemic, combined with data from the IMS on antiviral supply within the Member States, offer a unique opportunity to evaluate the association between NAIs and influenza mortality. The results of this analysis suggest that, after controlling for various differences between Member States, higher supplies of oseltamivir and zanamivir were significantly associated with reductions in H1N1 mortality from July 2009 to August 2010; namely, each 10% increase in kilograms of oseltamivir and zanamivir supply, per 100,000 people, was associated with 1.6% and 0.3% mortality reductions, respectively.

While this analysis demonstrates a strong association between NAI supply and pandemic H1N1 mortality over the course of the pandemic period, the observational nature of the data limits our ability to draw strictly causative conclusions. To better estimate the causal association, this analysis attempted to account for intrinsic differences between Member States that may bias the association between NAI supply and influenza mortality. For example, social and economic differences, as well as variations in expenditures on health, can independently account for differences in overall mortality between Member States, regardless of an influenza pandemic. Specific to influenza, an ecologic analysis of European countries found that mortality from pandemic H1N1 was negatively associated with gross domestic product and per capita governmental spending on health [18]. Additional differences in the susceptibility of a population to the pandemic H1N1 influenza virus may have biased an ecologic analysis, thus, data on at-risk populations, including the proportion of older individuals, degree of obesity, and pregnancy rate were considered. Further parameters, including public transportation usage and population density, were found to be related to hospitalization rates during the H1N1 pandemic in an ecologic analysis from California [20]. Population density was not found to be related to H1N1 mortality or NAI supply in this analysis, however. It is likely that population density may have a greater impact on total hospitalizations and the spread of influenza, but that different factors related to health spending and health care infrastructure have a greater impact on H1N1 mortality, as was found in this analysis and by Nikolopoulos, et al. [18] While every attempt was made to adjust for the many differences between Member States that could possibly bias an association between NAI supply and H1N1 mortality, the included co-factors were by no means exhaustive and several important confounders such as air quality or land use patterns – which may impact a population's susceptibility to influenza or exposure to infected livestock – may continue to bias the estimated main effect [18], [20]. Further, as with any ecological study, these associations may not be reflective of the individual-level association, and evidence from controlled studies is needed to evaluate the possibility of a causal relationship between NAIs and influenza mortality.

Apart from the above mentioned limitations, our analysis appears to be robust to several uncertain aspects of the included data. For example, the measure of pandemic H1N1 mortality used in the analysis was derived from the number of deaths with laboratory confirmation of pandemic H1N1 infection. Given lab capacity constraints, some Member States may be unable to verify all suspected pandemic H1N1 deaths. If a significant number of possible H1N1 deaths were not tested, national statistics reported to the WHO may underestimate the true mortality. To evaluate the potential impact of limited lab capacity on the models, sensitivity analysis was conducted on a data set adjusted to exclude all Member States not classified by the WHO as having full service National Influenza Centers. After excluding these Member States (Jordan, Saudi Arabia, and the United Arab Emirates), the association between NAI supply per capita and 14-month H1N1 mortality did not change substantially, suggesting that model results were not sensitive to possible underestimates in pandemic H1N1 mortality due to limited lab capacity.

Drug supply audits conducted by IMS cover international drug sales by retail pharmaceutical outlets and hospitals but may not be comprehensive due to the scope and political intricacies of national drug distribution. The corporation acknowledges this limitation and estimates the proportion of overall pharmaceutical drug supply that they are able to observe in each country-specific audit. Of the 42 Member States included in this study, IMS estimates that it has complete coverage of the overall pharmaceutical market for 23 (50%) of them and underestimates the true quantity obtained by those remaining. The median estimated percent coverage for the included Member States was 95% (IQR: 80.8%–100%). To correct for lower rates of coverage, the estimated drug supplies were increased from their reported value by the estimated percentage of coverage IMS had for each Member State based on a market survey. In sensitivity analysis, the corrected supply of NAIs demonstrated an even greater negative association with pandemic H1N1 mortality, although the results remained within the 95% confidence intervals of the coefficient estimates given by the original combined model.

Several other limitations exist that may impact the validity of our results. First, the results of this analysis depend on the assumption that the supply of NAIs reflects the total NAIs administered by each Member State. If, however, large quantities of NAIs were purchased and not distributed, or distributed but not administered during the pandemic period, the observed association may be an underestimate of the true effect that NAIs have on influenza replication and subsequent influenza mortality. For example, anecdotal evidence suggested that the UK may have stockpiled NAIs and only used a fraction of the supply for influenza treatment (C.R. Penn, personal communication). Because the IMS estimate of the drug supply market could not account for national stockpiling, the UK was excluded from the final set of 42 Member States. No other Member States were excluded for this reason.

Because many Member States ceased laboratory testing for all but critical cases of suspected H1N1, per recommendation from WHO, the data were restricted to the mortality rate (mortality among the general population) rather than the H1N1 case fatality rate (mortality among those with confirmed influenza infection) [21]. The impact is that our analysis is unable to assess whether some Member States had lower H1N1 mortality because they had fewer overall cases or because of other notable differences such as shorter times to hospitalization or to treatment with antivirals.

Finally, only 42 Member States, drawn from five of the six WHO Regions, had available data for inclusion in the analysis. As a consequence, the data set for analysis was primarily composed of developed Member States where national health and economic data were readily available. For example, of the Member States included in the data set, 26 are located in the European Region whereas no Member States from the African Region were included, thus limiting the scope and generalizability of the findings.

Treatment of symptomatic influenza with NAIs is a very effective intervention but only one of many needed to mitigate the severity of an influenza pandemic. Other key strategies, including vaccination, case isolation, school or workplace closure, and travel restrictions, will additionally be needed to have maximal reduction in influenza cases and mortality [5], [6], [7]. Furthermore, the NAI supply in a Member State is likely to be correlated with overall influenza pandemic preparedness. Therefore, the estimated relationship between antiviral supply and H1N1 mortality may be confounded by other preparedness activities, such as policies implementing social distancing, travel restrictions, and public health campaigns. We were unable to control for this confounding in our analysis. However, these non-pharmaceutical efforts may have impacted total H1N1 transmission over the pandemic period, whereas, pharmaceutical interventions, including NAI supply, most likely played a greater role in reducing mortality.

Limitations aside, this analysis demonstrates a statistically significant association between NAI supply and H1N1 mortality during the 2009 influenza pandemic at an ecological level and is consistent with many other publications that have demonstrated an impact of use of antivirals on the outcome of pandemic influenza in individual cases [3], [12], [22]. And while this analysis evaluated mortality from only one pandemic, recent findings suggest that the mortality seen during the 2009 H1N1 influenza pandemic was similar to that seen during seasonal epidemics and in the 1968 pandemic [23]. Thus, the association seen between NAI supply and H1N1 mortality may be generalizable to future influenza transmission seasons and pandemics similar to that of 2009. Furthermore, our analysis justifies the importance placed on efforts to treat influenza and may help policy makers and public health officials plan for future influenza pandemics.
